# Hospital Sunday Fund

**Published:** 1895-07-27

**Authors:** 


### HOSPITAL SUNDAY FUND.

THE Hospital Sunday Fund has now reached over £42,000, and it is felt that strenuous efforts should still be made to attain to the desired sum of £50,000. We publish two letters from the Lord Mayor and Sir Sydney Waterlow, which make further and powerful appeals on behalf of the Fund:—

TO THE EDITOR OF THE "HOSPITAL."

Sir,—As the Hospital Sunday Fund for the present year is about to be closed and distributed, I wish to make a final appeal for £4,000, which sum, if raised, will bring up the Fund, for the first time, to the gratifying total of £50,000. I feel that, if this were done, it would be an incentive for successful efforts in the future.

Though the collections have been numerous and satisfactory, and many generous donations have been received, the excitement and turmoil of the General Election, withdrawing large numbers from town, diverting people's attention from other matters, and resulting in a drawing-in of the charitable purse-strings, have had, unfortunately, a prejudicial effect on the receipts for Hospital Sunday. While the committee are grateful that the amount to be distributed is estimated at £46,000, I hope that, with yet one further effort, the handsome total of £50,000 may be reached, as London's offering this year to the maintenance of its hospitals and medical charities.

I shall be happy to receive, in the course of this week, any sums which may be addressed to me at the Mansion House.

I am, Sir, your obedient servant,
JOSEPH RENALS, Lord Mayor.

The Mansion House, London, July 22nd.

TO THE EDITOR OF THE "HOSPITAL."

Sir,—The growing importance of this organisation to the sick poor of London, and the great extent to which the hospitals, dispensaries, and convalescent homes that minister to more than a million poor patients lean upon the collections made on Hospital Sunday, must be my excuse for troubling you with these few lines.

For twenty-four years, as president, vice-president, and chairman of the distribution committee, I have taken the deepest interest and have devoted much time to the details of the working of this organisation. The annual receipts from all sources have risen from £27,700 in the year 1873 to £43,679 in the year 1894. For the present year we have already received £42,796. Several collections have still to be paid in. The total for this year already promised will probably not be less than £46,000.

In the face of this gradual improvement—which I think we are justified in regarding as the best evidence we could have of the confidence of the public in the action of the distribution committee—we are still without sufficient means to enable us to award to the several hospitals, &c., money enough to justify the managers in filling the 1,901 empty beds, the occupation of which would relieve such a very large number of sick poor, who now have to be turned away from the doors of the hospitals for want of funds.

It is very important the public should understand that the greatest care is taken to analyse the accounts and scrutinise the management of every institution making a claim for an award from the fund.

The managers of the several hospitals, &c., are now obliged to keep and render their accounts to the distribution committee on one uniform plan, which has received the full concurrence and approval of the hospital managers. This uniformity of account has greatly facilitated a proper comparison between the well-managed and ill-managed institutions. The accounts are required to be certified by a chartered or public accountant, thus insuring greater protection to the funded property and greater economy in the annual expenditure.

I mention these facts in order that the public may be satisfied that money given to the Hospital Sunday Fund is distributed with the greatest discretion and with a thorough knowledge of the relative needs and merits of each institution.

When the fund was first started under my presidency as Lord Mayor, in 1873, it was publicly stated by all those who were acquainted with the subject that £50,000 was the smallest sum a wealthy City like London ought to raise on Hospital Sunday. Since that time the population has so enormously increased that £100,000 would not meet the requirements.

Unfortunately, the legacies received by the various hospitals during 1894 are nearly £14,000 less than in 1893, and £139,668 less than in 1892. It should also be remembered that, owing to the increased facilities of locomotion, a much larger number of country patients are sent up to London hospitals to seek help from the many talented and skilled surgeons and physicians who settle themselves in London, and who give, gratuitously, so much of their valuable time and services to the relief of the sick poor.

A great effort is being made to raise the fund this year to £50,000, and many gentlemen of wealth have sent generous contributions to the Lord Mayor. The council is exceedingly anxious that the fund should this year reach £50,000, and that that sum should be regarded in future as the *minimum* for a year's collection.

The collection for this year will not be closed for a few weeks, during which time, I trust, through your kind help in giving publicity to this letter, the total for this year may be speedily raised to the amount stated.

I am, Sir, yours faithfully,
Sydney H. Waterlow, Vice-President.

July 22nd.

